# Is it possible to talk about violence climate in grassroots sport? A study on the psychosocial adaptations of young athletes

**DOI:** 10.3389/fpsyg.2024.1426900

**Published:** 2025-01-14

**Authors:** Juan González-Hernández, Manuel Gómez-López, Gustavo Carlo, David Manzano-Sánchez

**Affiliations:** ^1^Department of Personality, Evaluation, and Psychological Treatment, Faculty of Psychology, University of Granada, Campus Cartuja, Granada, Spain; ^2^Department of Physical Activity and Sport, Faculty of Sport Sciences, University of Murcia, Murcia, Spain; ^3^Cultural Resiliency and Learning Center, University of California, Irvine, Irvine, CA, United States; ^4^Faculty of Education, University of Almería, Almería, Spain

**Keywords:** grassroots sport, prosociality, victimization, aggressive behavior, competition, young athletes

## Abstract

**Introduction:**

There is a need for greater scientific attention to research on violence (e.g., insults, intimidation, beatings) in contexts where such behaviors are prevalent. The agonizing win-lose vision that oftentimes is characteristic of sports competition is not understood in the same way in grassroots sports as in professional sports. Although increasingly frequent, the federative systems for young athletes replicate professional competitions, and the agonizing win-lose vision and psychosocial agents that characterize sports competitions do not impact grassroots sports in the same way as in professional sports. The present study aimed to establish a predictive model of the influence of exposure to violence in initiation sports on peer social relations under competitive situations in young athletes.

**Method:**

Through a descriptive, non-randomized, and associative study, a sample of 503 young athletes (M_age_ = 14.76 1 ± 72 years; 54.80% girls) was recollected, belonging to different Spanish sports centres who completed instruments designed to identify their exposure to violence, prosocial and aggressive tendencies among peers, and competitiveness.

**Results:**

The results show that increased exposure to violence in sports amplifies those effects that excessive motivation for success and external influences, increases the likelihood of aggressive behaviors in young athletes (mainly in boys), while the emergence of prosocial skills in both boys and girls reduces aggressiveness and exposure to violent behaviors.

**Discussion:**

For this reason, to offer a more than relevant background in reducing the effects of excessive competitiveness in grassroots sport, scientific contributions on the protective efficacy of prosocial tendencies against the emergence of aggressive behavior. In addition, contemplating the sociological analysis of the proliferation of insults, harassment, and violent behavior (e.g., observed behaviors or victimization) experienced in sports at very early ages will allow, in a more applied vision, the convenience of designing more psycho-educational sport practice strategies (e.g., social skills integrated into sports action, fair play) both for young athletes and for those adults who accompany them (e.g., parents, coaches, managers).

**Conclusion:**

Addressing the effects of excessive competitiveness and violence in grassroots sports requires a comprehensive approach involving both sociological analysis and applied psycho-educational interventions.

## 1 Introduction

Despite the consideration of sport participation as a protective factor against aggressiveness and an enhancer of coexistence (Deliligka et al., 2017; González-Hernández et al., 2021), the existence of violence is understood as one of the aspects that hinder the natural development of learning not only at a sporting level, but also at a psychosocial level (Jones et al., 2017). The context of grassroots sports is increasingly like professional systems (Myer et al., 2015; Ticó, 2009), where figures as representative of young people as their parents (Dorsch et al., 2017), sports institutions (Jacobs et al., 2017), coaches (Lefebvre et al., 2019), and even among the same athletes among themselves (Stafford et al., 2013) exert their influence.

The young athlete, in the natural maturational process from childhood and adolescence, seeks to strongly identify positively with the groups in which he or she interacts socially encouraging more prosocial behaviors toward members of the social groups with which he or she identifies (Bruner et al., 2014; Donahue et al., 2009), and in case of perceiving threat to himself or herself or to such group, it helps more antisocial tendencies toward members of others to which he or she does not feel belonging (Zucchermaglio, 2005).

Competitiveness triggers such tendencies, sometimes motivated by cultures, languages or sports instructions (Kalina and Moustakas, 2024; Kellij et al., 2022). Boys’ tendencies have been associated with direct violence, fighting, and aggressive behavior, while girls have been linked with indirect violence (e.g., rumors, envy, victimization) (Bruner et al., 2017; Rees et al., 2015). In addition, perceiving and showing competence in performance tasks is part of the social cognitive evaluation that will facilitate efforts to integrate (e.g., respecting differences, accepting and perceiving oneself accepted by others) or withdraw (e.g., feeling guilty for failing or not being capable, avoiding showing oneself imperfect) socially (Boardley and Kavussanu, 2010; Kavussanu and Al-Yaaribi, 2021). Competing to achieve results is a justification on many occasions for violent behavior under arguments such as the “*value of winning*,” “*defending what is ours*,” “*thirst for revenge*” or equating sport to the epic war of battles (Houston et al., 2015; Kang et al., 2020). Thus, competitiveness is defined as a willingness to strive to meet a standard of excellence when comparisons are made in the presence of external evaluators (Martens, 1976). That is, it is constituted as an achievement behavior under psychological phenomena of social evaluation (e.g., pleasing, convincing, rivaling) (Cabrita et al., 2014; Daignault et al., 2023), also polarized with expectations to achieve success or avoid failure.

Assuming the unnecessary utility of violence in sports, its “nature” is understood in a completely different way than in other planes of life in general (Graupensperger et al., 2018). Conceiving it as a response to extremist positions (differentiating oneself from a norm) or pursuing individual identification with a reference group, could be two sides of the same coin in which violence joins sport (sometimes even justifying itself through the competitive) in concepts associated with a social response such as morality (Boardley et al., 2018; Hume and Wilding, 2015), social conflict (Bebber, 2015; Schneider and Eitzen, 2018), sports culture (Collins, 2016; Winands and Grau, 2018) or socioemotional maturation (Gini et al., 2014; Scholes-Balog et al., 2016). Still, very similar approaches apply to the violent climates to which young people are exposed by adults (Elliott and Drummond, 2017; Galán-Jiménez, 2018; Wright et al., 2013) or among peers (Cooley-Strickland et al., 2011; Monahan et al., 2015) in life in general (Chang et al., 2012).

Lang et al. (2023) mention that initiation practices and hierarchical dynamics such as “hazing” (mainly in team sports) create an environment conducive to the normalization of violence. Such practices can impact both boys and girls, exposing them to aggressive behaviors inside and outside the sports environment (Mooney et al., 2024). This type of initiation can perpetuate violent behaviors in social life beyond sport (e.g., machismo, aggressive behavior, mental health vulnerability). Furthermore, it has been observed that these violent dynamics include not only physical but also psychological and emotional abuse, which affects young people’s perception of what is acceptable in terms of behavior. Both boys and girls can be influenced by these contexts to develop patterns of aggression or social violence that carry over into other areas of their lives, including the school and social environment.

Studies on antisocial and prosocial behavior in sports (Gómez, 2007; Kavussanu, 2019), point out that violent responses in sports are the process resulting from the combination of negative interpretations associated with a frustration response, the vicarious and gratifying coexistence of previous violent episodes in close contexts, and the distancing of the negative understanding of the violent act (moral disengagement) (see Table 1). To this end, the strengthening and stability of prosocial (tendencies to help others) and educational (civility and community connectedness) traits are essential for personality and identity during childhood and adolescence and their protective effects against bullying, violence, and aggressive situations (Padilla-Walker and Carlo, 2014). Their protective effects against exposure to violence and competitiveness are described in that girls show higher levels of empathic concern, perspective-taking in the face of aggressiveness, violent situations, and prosocial behavior than boys (Carlo and Randall, 2001; Longobardi et al., 2019). On the other hand, although lower, levels of prosocial behavior are shown to be more stable until age 14 for boys, followed by an increase until age 17 and a slight decrease thereafter, while for girls, prosocial behavior increases until age 16 and then decreases slightly (Luengo Kanacri et al., 2013; Van der Graaff et al., 2018).

**Table 1:** Causes for violence in sports (adapted from Gómez, 2007).

Violence as the fruit of frustration	The one that occurs when, e.g., a match is lost, a play is missed or a provocation has been received, and aggression against the winning team takes place.
Vicarious reinforcement	Repetition of violent behavior that has already occurred by other sport agents (e.g., professional athletes, coaches, parents…) and has seen rewards (e.g., television or live).
Negative moral reasoning	It refers to the granting of “legitimacy” to perform an aggressive act. There is a moral departure from the social norm (whether written or unwritten).

To speak of exposure to violence is to raise the distinction between two fundamental issues: *direct exposure* (a person is a direct victim of a violent act) or *indirect exposure* (a person is a witness of violent behavior) (Buka et al., 2001; Ozer et al., 2017). Direct exposure to violence can come in different forms such as physical abuse (e.g., hitting, stealing, pushing), verbal abuse (e.g., name-calling, belittling), social exclusion (ignoring, marginalizing), sexual abuse (Finkelhor et al., 2015) or cyberbullying (Craig et al., 2020). The naturalness of living with insults, accusations, manipulations, belittling, or conspiracies in grassroots sports experiences, is a source of aggressive responses among peers with which first sports experiences are learned and faced (Kavussanu, 2019).

Similarly, exposure to violence among youth will entail the construction and learning of basic patterns of thoughts and attitudes (see Table 2) based on their role in the face of violent behaviors, where both *victimization* (Benedini et al., 2016; Turner et al., 2016) and the tyrannical behaviors of the violent (Dirks et al., 2017) are sources of significant and long-lasting socioemotional disturbances (Daignault et al., 2023; Fagan et al., 2014).

**Table 2:** Behavioral and emotional responses to exposure to violence.

	Victimization	Behaviors associated with the violent person
Reply behavioral	Lack of appetite, headache, general malaise, tiredness, feeling of suffocation, sleeping difficulties (nightmares or insomnia), social isolation, apathy and introversion, flight and avoidance behaviors, absenteeism (school, training…), uncontrolled crying, tremors, palpitations, restlessness, nervousness, suicide threats and attempts, etc.	Tyrannizing, intimidating, bragging, threatening, hitting, repeated participation in violent situations, sarcastic, defiant in front of adults and peers, physically strong, difficulties in socialization, manipulation of reality, conspiratorial, cleverly uses different ways for his actions, etc.
Reply socio-emotional	Fear or dread, anxiety, low self-esteem, alexithymia, depression, memory problems, difficulty in concentration and attention, irritability, permanent state of alertness, contradictory feelings of guilt or denial, pessimism, etc.	Low self-esteem, narcissism, aggressiveness, moral disengagement with situation and victim, difficulty regulating aggressive behavior, low empathy, low impulsivity, frustration, alexithymia.

N, sample size; Count; %: Percentage of cases over the total of said variable.

### 1.1 Exposure to violence in sports contexts and aggressive behavior profiles in young people

In most media sports (and especially in football), we experience worrying and increasing frequency, episodes of different types of violence between adults, mainly between their parents or coaches (Berns, 2017; McMahon et al., 2018), in front of referees (Walters et al., 2016) and between parents in the stands (Elliott and Drummond, 2017; Grasso et al., 2016) and although less, also among young athletes (Abajobir et al., 2017; Farrell et al., 2017; Vertommen et al., 2017). Too often, younger athletes are encouraged by adults (e.g., coaches, parents) to desire revenge. In school contexts (where the goals are to teach and educate young people), victimization has been directly and indirectly related to school violence through revenge motivation and failure avoidance (Benard et al., 2022; León-Moreno et al., 2019; Skrzypiec et al., 2018).

Besides, since in sports there may be actions that have adverse consequences for others (e.g., insults to referees, cheating, aggression, etc.), the negative consequences for the welfare of others fall within the moral sphere and are considered aggressive (Benard et al., 2022; Grugan et al., 2020; Stanger et al., 2017). Besides, male athletes are more physically, verbally, or non-verbally aggressive than women (Buka et al., 2001; Kimber et al., 2018; Vveinhardt and Fominiene, 2020). Men, not only as generators of violent situations but also in victimization, are more expressive than women (Albouza et al., 2020; Debowska et al., 2015). In addition, both boys and girls were exposed to violent contexts in the past and subsequently apply the same negative behavior in sports activities (Campbell et al., 2016; Eme, 2018; Finkeldey, 2019). Finally, it is observed that the environment that legitimizes aggressiveness may influence a greater openness toward aggressive behaviors, mainly in males (Turner et al., 2016; Wright et al., 2013).

Different studies have analyzed the role of exposure to violence in peer relationships in young’s sports, linking with the hyper-specialization and the mediatic social dimension (Brenner, 2016; Malina, 2010). Research has consistently identified certain sports contexts that are associated with a higher risk of exposure to violence. This is particularly evident in practices such as sports initiations, hierarchy-based intimidation, and hazing, which are more prevalent in team sports (Mountjoy et al., 2015). In individual sports, the close and ongoing coach–athlete relationship (Parent and Fortier, 2018) and the intense demands of competitive sports appear to create environments more susceptible to tolerating and exposing athletes to violence (Mountjoy et al., 2016). As Parent and Fortier (2018) summarize, elite athletes are at a heightened risk due to the intense nature of their training, which often involves separation from family or social support and constant proximity to an authority figure, typically a coach, who may exert control over various aspects of the athlete’s life, such as diet, equipment choices, sleep, training, and social relationships (Vertommen et al., 2016).

Despite the relevance of the variables described above, very few studies have analyzed the role of exposure to violence in peer relationships in young’s sports. A descriptive, transversal, non-randomized, and associative study, with a selective and survey methodology. As a first objective, the present work pretends to describe (according to gender) the relationships between exposure to violence, prosocial and aggressive behaviors, and competitiveness in young athletes. The second objective aims to establish a predictive model of the influence of exposure to violence in grassroots sports on social relationships (prosocial and aggressiveness trends) between peers in competitive situations in young athletes.

In this sense, and trying to answer the question research (inserted in the title), it is hypothesized that: (a) the exposure of youth to violent contexts (direct and indirect) in grassroots sport will amplify the positive relationship between competitiveness and aggressive behaviors, while prosocial behaviors will be a protective factor against both exposure to violence and aggressive behaviors among peers and, (b) greater aggressive responses will appear in boys and prosocial responses in girls, and boys will show greater competitive tendencies toward the pursuit of success, while girls will tend toward the avoidance of failure.

## 2 Methods

### 2.1 Participants

A non-experimental, relational, cross-sectional design with non-random sampling was used. In this research 503 adolescents with a mean age of 14.76 1 ± 0.72 years [boys: *N* = 237; *Mean* = 14.65 (SD = 1.72); girls: *N* = 266; *Mean* = 14.86 (SD = 1.77)] from different sports and club centres in different Spanish cities participated (see Table 3). Among other questions about their sports participation, participants reported a sports practice of 2.78 ± 0.23 days/week.

**Table 3:** Descriptive statistics of the participating subjects.

Variables		*N*	%	Chi-square
Sex	Male	237	47.12	0.096
	Female	266	52.88	
Days/week of physical activity and sport practice	0 days/week	58	11.53	0.000
	1–2 days/week	209	41.55	
	+3 days/week	267	53.08	
Federated sportsman	No	345	68.58	0.000
	Yes	158	31.41	
Physical Activity and Sport Practice Modality	Recreative physical activity	58	11.53	0.000
	Collective sports	184	36.58	
	Individual sports	106	21.07	
	Physical activity and health	155	30.81	
Type of sport (according to violence behaviors)	Contact and not mediatic sports (e.g., taekwondo, judo, volleyball, hockey)	104	20.68	0.014
	Mediatic and contact sports (e.g., soccer, boxing, basketball, handball)	103	20.48	
	Mediatic and not contact sports (e.g., tennis, track and field, handball, swimming)	83	16.50	
	General physical activity	213	42.34	
Subjective perception of competitiveness	Not competitive	123	24.45	0.000
	Somewhat competitive	139	27.63	
	Fairly competitive	147	29.22	
	Very competitive	94	18.69	

N, sample size; Count; %: Percentage of cases over the total of said variable.

### 2.2 Instruments

*Exposure to violence*. An adaptation to the sports contexts of the CEV questionnaire (Questionnaire of exposure to violence; Orue and Calvete, 2010) was used. It consists of 5 items adapted to the sports context, and describes two first-order exposures to violence orientations: 2 of direct exposure or victimization (“*How often have you been threatened with hitting you in your trainings and matches*”) 3 of indirect exposure where young people witness violence (“*How often have you observed how a person insulted or abuse another person in your trainings and matches*”). Also, a second-order index is described as Exposure to Violence. Each item was answered on a 5-point Likert-type scale, where 0 is *never* and 4 is *every day*. Cronbach’s alpha of the variables analyzed ranged between 0.75 and 0.83.

*Peer aggression*. The questionnaire Bullying, Fighting and Victimization [BFV; Bosworth et al., 1999, adapted to Spanish and named Peer Aggression Scale (EAP) by De Segredo et al., 2004] was administered. The instrument aims to assess aggressive behavior among young people (bullying) and describes four factors: (a) External Influences (“*I spoke badly about my peers*”), (b) Attitude Toward Violence (“*If I walk away from a fight I am a coward*”), (c) Prosocial Behaviors (“*I tried to make a new peer feel good in the group*”), and (d) Aggressive Behaviors (“*Others hit or threatened to hit a peer*”). The questionnaire version has a 35-item structure on a Likert-type scale from 1-*totally disagree* to 5-*strongly agree*. Cronbach’s alphas ranged from 0.76 on attitudes toward violence to 0.83 on aggressive behaviors.

*Competitiveness*. For the present research, the Competitiveness-10 questionnaire (Martens, 1976), Spanish version adapted by Remor (2007) was used to assess adolescents’ sports competitiveness. Participants indicate how competitive they feel by responding to 10 items [6 success motivation items (“*The harder the challenge, the better I perform*”) and 4 failure avoidance items (“*I worry about what others will think of my performance*”)]. Responses were collected on a Likert-type scale with values between 1 (*rarely*) and 3 (*always*). Cronbach’s alpha showed 0.84 for success motivation and 0.82 for failure avoidance.

### 2.3 Procedures

First, the response templates for the assessment instruments were developed using the Google Form application. The administration of the questionnaires was carried out through electronic devices provided by the researchers under the criteria of “young people practicing federated sports after school hours.” Previously, informed consent had been provided to sports technicians and coaches in Clubs and sports federations (by convenience through personal contacts of the investigators) so that the young people could send them to their parents and facilitate the administration of questionnaires with their prior authorization. The questionnaires were administered to the young people in groups, without the presence of their coaches, guaranteeing anonymity and confidentiality of the data obtained. The latter could ask the research staff any questions that arose when completing the data. The methodological protocol of this study was approved by the Ethics Committee of the University of Granada (1726/CEIH/2020), following the Declaration of Helsinki (World Health Organization, 2015) and ethical guidelines of the American Psychological Association (APA).

### 2.4 Data analysis

Statistical analyses were conducted using the Statistical Package for the Social Sciences (SPSS. 25 and AMOS. 26. IBM Co. Armonk, NY). The data of the variables analyzed were described through central tendencies (mean, standard deviation, skewness, kurtosis, and variance). First, a MonteCarlo data simulation (Muthén et al., 2017) was performed to obtain a more reliable and stable estimate of the sample size (*p* < 0.05; d < 0.20) (Cohen, 2013; Gignac and Szodorai, 2016). The normality of the variables analyzed was calculated through the Kolmogorov–Smirnov test, except in variables with <50 subjects in which the Shapiro-Wilks test was used. All the variables analyzed reported having a parametric distribution (*p* > 0.95). Differences between the variables analyzed as a function of sex were performed using the *T*-test for independent samples (*p* < 0.05; bootstrapping = 5,000). Subsequently, Pearson correlation analyses were conducted (controlling for gender and sports practice), to determine both bidirectional relationships between peer aggression tendencies, exposure to violence, and competitiveness.

Moderating effects of gender on the relationships between exposure to violence and competitiveness with aggressive tendencies were tested using a nested, multigroup comparisons model (Byrne, 2013) under the guidelines of structural equation modeling (SEM). Also, invariance was examined through a multigroup structural model (focused on CFI and *χ*^2^/df), is looking to test predefined data sets to identify significant differences between group-specific parameter estimates (Bentler and Wu, 2002; Cheah et al., 2020). In the alternative condition, constraints were added, forcing the parameters of interest to be equal in the two groups. In this approach, if the model with equality constraints provides a significantly worse fit to the data than the unconstrained model, the constrained parameter is not equivalent between the groups (i.e., gender), suggesting an interaction or moderating effect of the grouping variable.

## 3 Results

Table 4 shows the descriptive analysis of the variables analyzed. The distribution, using the statistical calculations of skewness, kurtosis, and variance, allowed us to observe that the highest means and variances were found in prosocial behaviors and external influences for aggression among peers, and exposure to violence on sport [observation, victimization, and exposure (global index)].

**Table 4:** Comparative *t*-means of the variables analyzed in this research, according to sex.

Variable	Subvariables	M (SD)	V	A	K	Male		Female		*t*	*p*	*d*
						*M*	*SD*	*M*	*SD*			
Peer aggression	CP	4.14 (0.79)	0.63	−1.33	2.08	3.97	0.86	4.30	0.70	3.71	**	−0.43
	IE	2.91 (0.57)	0.33	−0.26	0.26	2.87	0.59	2.94	0.56	3.46	n.s.	−0.12
	AV	2.67 (0.69)	0.48	−0.63	−0.07	2.73	0.66	2.63	0.71	3.27	n.s.	0.09
	CA	1.66 (0.58)	0.33	1.34	2.40	1.91	0.59	1.35	0.54	4.02	**	0.46
Exposure to violence in sport	Ovs	1.42 (0.99)	0.97	0.43	−0.36	1.97	1.10	1.23	0.83	3.75	**	0.45
	Vvs	0.48 (0.69)	0.48	1.64	2.92	0.78	0.81	0.25	0.45	6.79	**	0.83
	Evs	0.95 (0.75)	0.56	0.87	0.59	1.22	0.88	0.74	0.54	5.57	**	0.67
Competitiveness	MotE	2.57	0.39	0.15	−0.23	2.73	0.34	2.44	0.38	3.58	**	0.80
	AvF	1.83	0.46	0.21	0.35	2.07	0.41	1.89	0.50	4.09	*	−0.26

^*^p < 0.05 and ^**^p < 0.01. M, Means; SD, Standard Deviation; V, Variance; A, Asymmetry; K, Kurtosis; d, Cohen index; Ovs, Sports practice violence observation; Vvs, Sports practice violence victimization; Evs, Sports practice violence exposure (global index); CP, Pro-social behaviors; IE, External influences; AV, Attitudes toward violence; CA, Aggressive behaviors; AvF, Avoidance of failure; MotE, Success Motivation.

### 3.1 Differential analysis by gender

Besides, Table 4 describes the mean difference analysis of the variables analyzed, according to the sex of the participants. Regarding peer aggression, statistically significant differences were observed in prosocial behaviors (*p* < 0.01) with higher values in girls and aggressive behaviors (*p* < 0.01) with higher values in boys. Specifically, significant differences were observed in both observation (*p* < 0.01) and victimization (*p* < 0.01), and consequently in exposure to violence (*p* < 0.01). Finally, as a function of competitiveness, statistically significant differences were shown in the motivation for success (*p* < 0.01) with higher values in boys, while the values were statistically significant and higher in the avoidance of failure (*p* < 0.02) also in boys.

### 3.2 Correlation analysis, according to gender

In the coexistence relationship between the variables under study (see Table 5), controlling for the gender differences already demonstrated, it was found that exposure to violence and competitiveness in youth sports contexts is related to the appearance of aggressive tendencies in both girls and boys. Positive linear relationships between violence observation and victimization (exposure to violence as well, correspondingly), were shown to be positively significant with attitudes toward violence (*p* = 0.00) and perceptions of external influences (*p* = 0.00), and aggressive behaviors (*p* = 0.00), whereas the links are negative if prosocial behaviors are higher (*p* = 0.00).

**Table 5:** Relational analysis of the variables under study in this research, according gender.

		1	2	3	4	5	6	7	8	9
Exposure to violence in sport	Ovs	–	0.56^**^	0.71^**^	−0.25^**^	0.35^**^	0.33^**^	0.32^**^	0.13^*^	0.19^**^
	Vvs	0.62^**^	–	0.75^**^	−0.32^**^	0.33^**^	0.41^**^	0.28^**^	0.16^*^	0.26^**^
	Evs	0.59^**^	0.66^**^	–	−0.43^**^	0.46^**^	0.62^**^	0.54^**^	0.19^*^	0.28^**^
Peer aggression	CP	−0.36^**^	−0.48^**^	−0.52^**^	–	−0.34^**^	−0.46^**^	−0.57^**^	−0.28^**^	0.34^**^
	IE	0.25^*^	0.31^**^	0.39^**^	−0.45^**^	–	0.38^**^	0.44^**^	0.27^**^	0.26^*^
	AV	0.19^**^	0.24^**^	0.56^**^	−0.52^**^	0.28^**^	–	0.31^**^	0.24^*^	0.29^**^
	CA	0.28^**^	0.20^**^	0.36^**^	−0.64^**^	0.32^**^	0.26^**^	–	0.29^*^	0.32^**^
Competitiveness	EvF	0.11^*^	0.12^*^	0.16^*^	−0.39^**^	0.33^**^	0.19	0.20^*^	–	0.17
	MotE	0.20^**^	0.23^**^	0.24^**^	0.56^**^	0.21^*^	0.19^**^	0.23^**^	0.12	–

*p < 0.05 and **p < 0.01. Correlations for boys (*n* = 237) are presented above the diagonal and correlations for girls (*n* = 266) are presented below the diagonal. Ovs, Sports practice observation; Vvs, Sports practice violence victimization; Evs, Sports practice violence exposure CP, Pro-social behaviors; IE, External influences; AV, Attitudes toward violence; CA, Aggressive behaviors; AvF, Avoidance of failure; MotE: Success Motivation.

Finally, although the correlation indices are higher in boys than in girls, competitiveness showed also positive relationships with exposure to violence and aggressive trends, both in success motivation (*p* = 0.00) and avoid failure (*p* < 0.01).

### 3.3 Predictive analytics

Before testing for differences in the estimates of exposure to violence to aggressive tendencies, we tested models in which the paths from demographic covariates to outcomes and stability in the coefficients of aggressive tendencies (e.g., frequency of physical activity) were restricted to be equal by gender. These restrictions did not lead to a worse-fitting model. Thus, a model with paths (boys vs. girls) from the competitiveness variables to the obtained aggressive tendencies provided a good fit to the data [X^2^ (*df* = 29, *N* = 503) = 43.11; *p* = 0.09, CFI = 0.994, RMSEA = 0.027]. In both the first model (to boys) [X^2^ differential (*df* = 2; *N* = 503) = 6.01; *p* < 0.01] and the second (to girls) [X^2^ differential (*df* = 2; *N* = 503) = 8.05; *p* < 0.03], significantly adequate fits were achieved.

**Figure 1:**
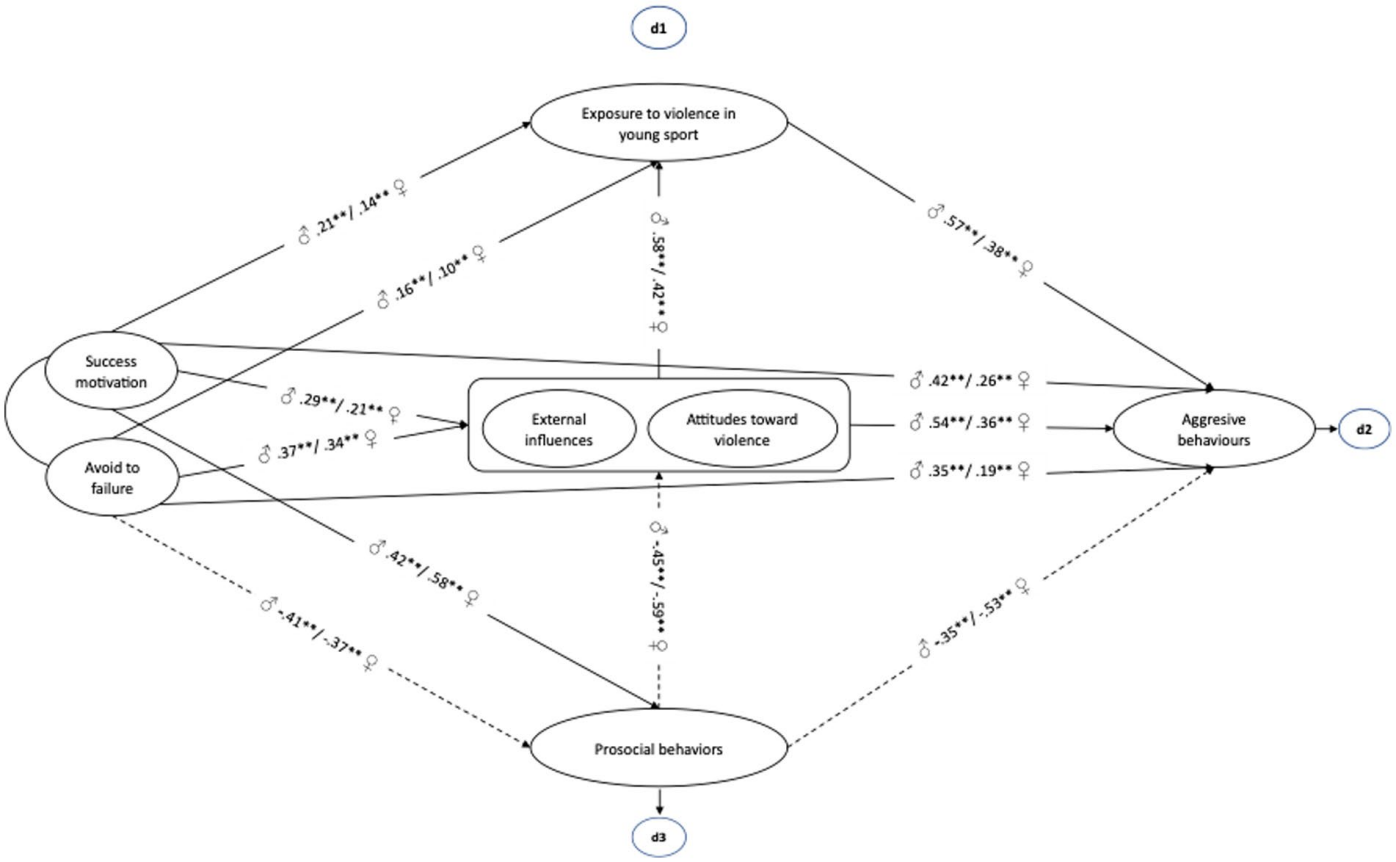
Path analysis model in young athletes.

Figure 1 shows the path model (moderation) showed that both competitiveness (*p* = 0.00) and aggressive tendencies (a combination of external influences and attitudes toward violence) (*p* = 0.00) enhance their predictive power through exposure to violence in sport (*p* = 0.00) for the occurrence of aggressive behaviors in young athletes. However, prosocial behaviors exert a protective value for aggressive behaviors in young athletes (*p* = 0.00) and exposure to violence in sports (*p* = 0.00). A non-significant difference was found between the unconstrained and the constrained model [ΔS-B X^2^ = 69.32; *df* = 10; *p* > 0.05 (ns)]. This result supports the structural invariance of the model in both genders, which adds further generalization and applicability to the model.

## 4 Discussion

The excessively competitive orientation of grassroots sports encourages an excessive tendency to the appearance of violence (e.g., verbal, physical, intimidating) as a strategy for achieving positive results at an early age. While there is an extensively documented association between exposure to violence and its relevance to negative responses in competitive grassroots sports settings (e.g., aggression, frustration) (Stanger et al., 2017), research has focused much less on the impact of exposure to violence on tendencies toward greater or lesser aggressiveness among peers, and existing research has not clarified whether there are gender differences in the effects of exposure to violence and aggressive behaviors. It is important to study gender differences in aggressive tendencies of exposure to violence in grassroots sports contexts, as well as in the competitive orientation of violence, to advance understanding of its relevance for prevention and intervention. This study, through a cross-sectional study, examines gender differences in the effects of exposure to violence on aggressive tendencies among peers in a large and diverse sample of young athletes.

The aims of the present study pursued different purposes in its design are to identify an individual (behavioral, cognitive, and attitudinal) and contextual (observation and victimization to violence) responses and perceptions of competitiveness, according to gender, and to explore direct and predictive connections between exposure to violence, competitiveness, and the occurrence of aggressive behaviors among peers, taking into account gender as a moderator.

### 4.1 Exposure to violent contexts as a source of aggression among peers in youth sports

Fulfilling the stated hypotheses 1, both the linear model and the predictive model offer significant evidence of a relevant increase in aggressive behaviors for those adolescents who are more exposed to violence. Exposure to violence is one of the causes of human violence and aggression because behaviors can be learned by both imitation and direct reinforcement (Cooley-Strickland et al., 2011; Skrzypiec et al., 2018) as observational or vicarious learning (Grasso et al., 2016; Orue and Calvete, 2010).

In addition, adolescents in permanent contact with violence in each context predispose to be so both in that context and in others with similar functioning (Contreras and del Carmen Cano, 2016). In this sense, the present study was adjusted to this hypothesis in the sports context, where young athletes who observe or feel victims of any type of violence, feel more permeable to external influences toward violence, show lower prosocial responses, and finally, show greater aggressive responses in relationships with their peers. However, significant relevance of competitiveness was observed, contrasting with other studies which highlighted that the perception of achievement (mainly an excessive success motivation), could be a source of sporting frustration and resort to aggressive behaviors as mechanisms of social action in adolescent athletes (Houston et al., 2015; Vertommen et al., 2017).

### 4.2 Gender differences in sports contexts

Partially following the hypothesis 2, in a statistically significant way, boys were more exposed to violence than girls in all the contexts considered in the model, except in the family context and the observation of violence on television. Different studies confirm these results in general contexts, highlighting the association of the male gender with greater contact and victimization with violence. Díaz-Atienza et al. (2004) similarly found a greater exposure to violence in boys in a study with 410 students between 12 and 17 years old, while Zegarra et al. (2009) reported greater exposure to violence both in the role of bully and the bullied, in boys within a sample of 641 adolescents between 12 and 16 years old. More recent studies have indicated that boys show greater victimization and exposure to violence, associated with moral disengagement in their everyday contexts (Romera et al., 2015). In sports contexts, recent studies highlight those that point out the same trend in favor of boys (Parent and Fortier, 2018) and other more specific ones where it is described that boys are exposed to physical violence, while girls are more exposed to both psychological and sexual violence (Vertommen et al., 2016).

Adjusted to the hypothesis 3, the results showed that girls showed significantly higher values in prosocial behaviors, while boys presented statistically higher values in aggressive behaviors. Data corroborate studies around antisocial and prosocial behaviors in adolescent samples (Farrell et al., 2017; Kraft and Mayeux, 2018). In sports contexts, studies in adolescent samples have shown that girls show much more prosocial orientations regardless of their competitive vs. non-competitive orientation (González-Hernández and Martínez-Martínez, 2020), whereas boys’ antisocial behaviors have been linked to an ego orientation, reinforced by aspects such as reduced mental toughness (Boardley and Kavussanu, 2010), low social cohesion (Bruner et al., 2014) or lower empathy (Stanger et al., 2017), although the vast majority emphasizing significant moral disengagement (Graupensperger et al., 2018; Kavussanu, 2019).

In terms of gender, statistically significant differences were detected in favour of boys in the motivation toward success and favour of girls in the motivation toward the avoidance of failure. Both in studies with the general population (Anderson et al., 2008; Dreber et al., 2014) and studies with sports samples, the results are similar (Vaz et al., 2014; Prieto Andreu, 2016).

Honestly, it is necessary to point out some limitations that are conceivable for the results obtained in the present study, and that could be considered with a view to its replicability. Firstly, the description of data obtained under a cross-sectional design, assuming the difficulty of arguing conclusions about causal effects. On the other hand, although the sample size is adequate for a study of these characteristics taking into account certain limited access (need for permits, centres, and places of contact), the data refer to a particular sample of Spanish young people, so the results could be much more generalizable to a larger sample of adolescents. The use of self-reports (one of them, with an adequate adaptation) could be a handicap when it comes to aspects such as comprehension (although a researcher was always present during data collection), as well as with other aspects such as social desirability.

Even so, and considering the above, the design and results of the present study allow us to guarantee its reliability and suitability. At the same time, information has been complemented and expands on what previously existed in the scientific literature on exposure to violence and provides new avenues for studies that describe the reality of grassroots sport in these aspects (e.g., studies according to age groups, competitive levels, sport modalities; mediation analysis). At the same time, to reinforce the limitations of the cross-sectional approach, it is necessary to complement the present study with long-term proposals, which will allow for a more in-depth study of the effects of exposure to violence on hostile or aggressive responses, as well as on the protective effects of prosocial behaviors under a developmental perspective. In addition, it would be necessary to include the perceptions of other representative and influential figures in sports contexts (e.g., coaches, parents, physical education teachers), to configure with greater amplitude, models of contextual influence and hostile responses in young athletes.

## 5 Conclusion

Throughout this article, we have highlighted how adolescents perceive violence in different contexts, and how these perceptions are related to competitiveness and the emergence of behaviors and attitudes towards aggressiveness. Trying to answer the question of whether or not there is violence in grassroots sport, and taking into account that there are contexts where more media sports magnify the determinism with which results, achievements and successes/failures are experienced, different tendencies about to violence exposure and psychosiciological adaptations in sport youngers have been studied.

Focusing the data on perceptions toward sports practice, considering certain behaviors or psychological responses as “natural” and “normalized,” with which we live in grassroots sport, is a high risk for the emergence of hostility toward others in young athletes., Observed, performed or perceived among young athletes and the adults around them, attitudes and behaviors will increase (e.g., intolerance, vindictive or envious tendencies, justification of violence as a means to achieve goals) and they will interpret violence as part of those behaviors integrated in learning and focused on sport achievements.

Reciprocally, the emergence and learning of aggressive behaviors and attitudes create hostile environments for themselves and others. Hence the importance of psychoeducational interventions that allow the training of prosocial skills (e.g., empathy or altruism) and social cooperation (e.g., solidarity). Along with this, acquiring and/or enhancing aspects of emotional self-regulation (e.g., forgiveness, compassion) and behavioral (e.g., fair play, help), will allow the emergence of protective factors against responses of frustration or humiliation in sporting experiences.

## Data Availability

The raw data supporting the conclusions of this article will be made available by the authors, without undue reservation.
